# Medical education: The need for uniform standards!

**DOI:** 10.4103/0970-1591.65378

**Published:** 2010

**Authors:** Nitin Kekre

**Affiliations:** Department of Urology, Christian Medical College, Vellore - 632 004, Tamil Nadu, India. E-mail: editor@indianjurol.com

During the last few years, the Urological Society of India has made significant efforts in improving urological education, and setting up of a separate education cell was the right step. The education cell has organized various teaching programmes and the “Mock Examination” is probably the most popular one, for obvious reasons. Most of the candidates want to qualify and get accredited as a “Qualified Urology Consultant.” And, this is reflected in the improved pass percentage in the National Board Examinations. But, one must pause and think about the ultimate objective. Do we want the future generation of Indians to be treated by Urologists who could successfully acquire the qualification (be MCh or DNB) but may not have been adequately trained in their surgical skills? Would we be happy to fly with a pilot who has obtained the right qualification and has been trained on a virtual trainer but has never flown in a real-life situation? The need for uniform, minimum basic standards of training cannot be over-emphasized. As a learned society, we should be able to assure the public in general that a qualified urologist is academically sound and is well trained in handling basic urological problems in a competent fashion. This can only be achieved by developing a central body, which can regulate all higher medical education in the country. It would require a coordinated effort of all the bodies involved in medical education, which would then govern and control the residency programmes in this country. Accreditation should be done by the centralized board that would guarantee the minimum standards. We have, in our country, examples of such institutions like the IITs, IIMs, NIFTs etc., which have managed to produce professionals of high standards. There is an on-going debate as regards who should control the medical education: should it be the Health Ministry or the Ministry of Human Resource? What should be the role of State versus Center? There is a lot being written about it in the general press, more so after the recent Medical Council of India scandal. But, surprisingly, the important stake holders have been silent. Especially the societies like IMA and different medical or surgical associations. I personally feel that this is the right time to speak out and ensure that our views are heard. We should make sincere efforts to clean up the mess of medical education. Recently the BAR Council of India has come out with the proposal that all law graduates in the country would have to undertake a national qualifying exam before they can practice law. Similar mechanism would have to be put in place regarding medical practice. The health ministry can be involved in the delivery of healthcare but the medical education should remain under an independent non-governmental organization. The composition of this organization should be merit based and should include people who have extensive experience in teaching and research. But, education must remain above all this. I feel that the notion of Mr. Kapil Sibal, Honorable Minister of Human Resource, about primary education: one nation, one board, one exam, should also be applicable to all levels of education.

I am thankful to Mr. Andrew Thorpe, Consultant Urologist, Freeman Hospital, Newcastle Upon Tyne, UK, for guest editing the symposium on Urinary Incontinence & Over Active Bladder. During the last decade, significant developments have occurred in the management of overactive bladder, and urinary incontinence I am sure that this symposium will definitely enhance our understanding of this common trouble.

